# Expression of intra-tumoral necrosis-associated cytokine pattern correlated with prognosis and immune status in glioma

**DOI:** 10.3389/fnmol.2023.1117237

**Published:** 2023-07-03

**Authors:** Hongtao Zhao, Jiawei Dong, Jiheng Zhang, Nan Wang, Zhihui Liu, Xiuwei Yan, Fang Wang, Hang Ji, Shaoshan Hu

**Affiliations:** ^1^Cancer Center, Department of Neurosurgery, Zhejiang Provincial People’s Hospital, Affiliated People’s Hospital, Hangzhou Medical College, Hangzhou, Zhejiang, China; ^2^Department of Neurosurgery, West China Hospital, Sichuan University, Chengdu, China

**Keywords:** glioma, intra-tumoral necrosis, cytokine, RNA sequencing, immune status

## Abstract

Intra-tumoral necrosis (ITN) is reported to be an independent prognostic factor in glioma. However, knowledge of ITN is mainly limited to pseudopalisadwe, while its other aspects were neglected. Therefore, a deeper understanding of ITN could be valuable for understanding its exact role in glioma. The only reliable ITN model was time-dependently achieved with the GL261 syngeneic mouse model. The ITN-associated expression pattern was enriched from RNA sequencing. TCGA glioma samples were clustered into a high-expression group (HEG) and a low-expression group (LEG) based on their pattern and their association with prognosis, clinical status, immune status, and therapeutic responsiveness were compared. Mouse glioma with ITN demonstrated invasive histology. Cytokine signaling was significantly enriched in necrotic mouse glioma compared with non-necrotic glioma tissues. Nine pro-inflammatory (IL6, PPBP, IL1A, TNFSF11, CXCL11, CXCL9, CXCL10, CXCL3, and CCL8) and two anti-inflammatory cytokine (IL1RN and IL10) genes were found to be related to ITN-associated cytokine patterns. Comparative analysis showed that HEG had a significantly shorter survival time, five differentially distributed clinical statuses, more infiltrated immune cells, greater expression of immune checkpoints, and better therapeutic responsiveness than LEG. In conclusion, the ITN-associated cytokine pattern is characteristically expressed in glioma with ITN and might indicate necrosis missed in histology diagnosis. Its expression pattern could predict the prognosis, tumor grade, immune status, and therapeutic responsiveness of glioma patients.

## 1. Introduction

The circulatory system delivers nutrients and oxygen to all cells in the body, and a shortage in blood supply induces infarction in normal tissues and intra-tumoral necrosis (ITN) in solid tumors (Mamun et al., 2009). ITN was shown to be an independent factor for poor prognosis in various malignant solid tumors and consistently associated with other high-risk factors (Richards et al., 2011). Regarding glioma, the most prevalent primary malignant tumor in the central nervous system, ITN has been reported to be an adverse prognostic factor in astrocytoma (Nelson et al., 1983), oligodendroglioma (Miller et al., 2006) and glioblastoma (GBM) (Barker et al., 1996); thereby, significantly reducing the survival of patients with increasing necrosis ratio (Hammoud et al., 1996). In addition, the survival of patients with extensive histological necrosis was at least half that of patients with limited necrosis (Louis et al., 2016). Thus, ITN is considered to be strongly related to glioma malignancy.

ITN has been mainly investigated in GBM owing to its unique histological feature, pseudo-palisades, which is a palisade-like hypercellular zone surrounding necrotic foci (Wippold et al., 2006). Interestingly, it was reported that the accumulation of cells resulted from cell migration rather than proliferation. Pseudo-palisades are composed of hypoxic cells that possess a higher ability to migrate and invade after ischemic events (Brat et al., 2004; Mamun et al., 2009). However, ITN comprises not only apparent pseudo-palisades but also central cell death, accompanied by the loss of plasma membrane integrity and the release of intracellular molecules (Marshall et al., 2014) that combine on the receptors of adjacent cells to regulate their status. Thus, further recognition of ITN in glioma relies on investigating these molecules and their potential effects.

Among the uncountable molecules released by a necrotic cell, several were confirmed to be tumor-promoting. Many damage-associated molecular patterns (DAMPs) were reported to be tumor-promoting in various cancers (Hernandez et al., 2016). High mobility group box 1 (HMGB1), one of the DAMPs, was reported to induce epithelial-mesenchymal transition (EMT) of glioma cells (Li et al., 2019). However, necrosis release a variety of molecules, and the initial concentration of these molecules cannot be characterized. Notably, the response of adjacent cells results from the activity of all molecules released in ITN. However, investigations on ITN-related changes in glioma have been limited to observing cellular reactions associated with ITN through naturally occurring INT *in vivo*. Encouragingly, non-necrotic and necrotic stages were observed in the GL261 syngeneic mouse glioma model (Cha et al., 2003), making it the only reliable model to characterize the changes associated with ITN in glioma.

To describe ITN-related changes, we compared the differences between non-necrotic and necrotic glioma histology and transcriptome. Gene enrichment analysis was conducted to determine the underlying gene expression pattern, verified in an experimental ITN model and GBM patient data, and characterized by histological necrosis in the TCGA database. Further, by clustering TCGA glioma samples with the ITN-related expression pattern into a high-expression group (HEG) and a low-expression group (LEG), we analyzed the differences in prognosis, clinical status, immune status, and predictive therapeutic outcome between the two groups.

## 2. Materials and methods

### 2.1. Mouse orthotopic syngeneic glioma model

The GL261 cell line was cultured with DMEM supplemented with 10% fetal bovine serum (B.I.). Upon reaching 70% confluence, the cell was trypsinized, centrifuged, and resuspended in ice-cold PBS without Ca2+ and Mg2+ at a concentration of 10^5 cells/2 μL in a 200 μL sterile tube. Female 6–8-week C57BL/6 J mouse was anesthetized with Isoflurane and fixed on the stereotactic instrument (RWD, 68861 N) supplemented with a mouse head holder (RWD, 68057). Two microliter GL261 cell solution was injected to a depth of 3 mm, 0.5 mm anterior, and 2 mm right to bregma. This procedure was performed with a 2 μL micro-syringe (Hamilton, 65,459–01) assisted by a syringe pump (KDS, legato 130) at a rate of 1 μL/min. Tumor-bearing mice were sacrificed on days 10 and 20, and their brain tissues were used for histology and RNA sequencing analysis. The animal experiment was approved by the Animal Ethical and Welfare Committee of Zhejiang Provincial People’s Hospital (No. A202100014).

### 2.2. Histology of mouse glioma

The brain of the mouse model on day 10 (*n* = 3) or 20 (*n* = 3) was sliced into 2 mm coronal slices and fixed with 4% paraformaldehyde, de-hydrated with gradient alcohol, de-alcoholed with environment-friendly dewaxing fluid, and embedded with wax. Then, a 4 mm tissue slide cut was performed using a Rotary Microtome (LM2016, Leica) for H&E staining. The mounted slide was scanned with a digital slice scanner (KF-PRO-120, KFBIO).

### 2.3. Whole tumor mRNA sequencing

Glioma of the mouse model on day 10 (*n* = 3) or 20 (*n* = 3) was separated from the tumor edge under a light microscope and frozen at-80°C. Total RNA was extracted, and genome-wide transcriptomics analysis was conducted (LC-BIOTECHNOLOGIES (HANGZHOU) CO., LTD). The differentially expressed genes (DEGs) of necrotic glioma (NG) versus non-necrotic glioma (NNG) were selected with|Log2 (fold change)| > 1 and *q* < 0.05 using the R package DESeq2.^1^

To perform whole tumor mRNA sequencing, total RNA was isolated and purified from samples using TRIzol according to the manufacturer’s instructions. The quantity and purity of the total RNA were assessed using NanoDrop ND-1000, while RNA integrity was evaluated using Bioanalyzer 2,100. Samples with a concentration greater than 50 ng/μL, an RIN value greater than 7.0, and total RNA greater than 1 μg were considered suitable for downstream experiments. mRNA with poly (A) tails was selectively captured using oligo (dT) beads via two rounds of purification. The captured mRNA was then fragmented at high temperature, and cDNA was synthesized using reverse transcriptase. *E. coli* DNA polymerase I and RNase H were used for double-stranded synthesis, converting the DNA–RNA duplex into a double-stranded DNA. dUTP solution was incorporated during this process to create blunt ends. A single A nucleotide was added to each end of the resulting double-stranded DNA, allowing for adapter ligation to the ends of fragments with T nucleotide overhangs. The double-stranded DNA fragments were then size-selected and purified using magnetic beads. After digestion with UDG enzyme, a library of fragments ranging in size from 300 bp ± 50 bp was generated using PCR. Finally, the library was subjected to paired-end sequencing using Illumina Novaseq™ 6,000 according to standard protocols, with a PE150 sequencing mode.

### 2.4. Gene enrichment analysis

Up-regulated and down-regulated DEGs were determined using Gene Ontology (GO) enrichment analysis, including biological process (BP), molecular function (MF), and cellular component (CC) (*p* < 0.01, *q* < 0.05, adjusted *p* by BH) in Hiplot.^2^

### 2.5. Verification of enriched genes

In mice, genes were verified by qPCR. Whole tumor RNA was extracted using the MolPure® Cell/Tissue Total RNA Kit (Yeasen, 19,221), transformed into cDNA with Hifair® III 1st Strand cDNA Synthesis SuperMix for qPCR (gDNA digester plus) (Yeasen, 11,141), amplified with Heff UNICON® Universal Blue qPCR SYBR Master Mix (Yeasen, 11,184), and quantified with the Real-Time PCR System (Applied Biosystem, 7,500) according to protocols from each manufacture. The primers used are presented in Supplementary Table S1.

### 2.6. Information on human glioma

Transcriptomic data (normalized FPKM), pathological data (status of IDH, 1p/19q, MGMT promotor), and clinical data (age, gender, and survival information) of human glioma were retrieved from The Cancer Genome Atlas (TCGA) database.^3^ After excluding samples without survival information, a total of 616 glioma samples were retained for further analysis. The R package “limma” was used to remove the datasets’ batch effect.

### 2.7. Consensus clustering

The k-means approach was used to recognize ITN-associated cytokine patterns with the expression of the selected cytokines. The consensus clustering algorithm was used to decide the number and stability of clusters using the “Consensus Cluster Plus” package. In addition, 1,000 iterations were conducted to ensure the robustness of the categorization (Wilkerson and Hayes, 2010).

### 2.8. Analysis of immune status

The content of the immune and stromal cells in the TME of each sample was assessed using the “ESTIMATE” algorithm and expressed into the estimated score, tumor purity, immune score, and matrix score (Yoshihara et al., 2013).

Immune cell infiltration was quantified with the ssGSEA algorithm. The characteristic genes for each type of immune cell were determined as previously reported by Jia et al. (Jia et al., 2018). The abundance of each infiltrated immune cell is presented as the ssGSEA score distributed from 0 to 1.

Tumor immunophenotype profiling (TIP)^4^ is a tool providing analysis and visualization of the extent of tumor-infiltrating immune cell and anti-tumor activity across the seven-step Cancer-Immunity Cycle (Xu et al., 2018). TIP was used to evaluate the differences in Cancer-Immunity Cycle between the necrotic and non-necrotic groups.

### 2.9. Prediction of therapeutic responsiveness

Chemotherapy reactivity was assessed with the “pRRophetic” package based on the Genomics of Drug Sensitivity in Cancer (Geeleher et al., 2014). The half maximum inhibitory concentration (IC50) was estimated using ridge regression, whereby a lower IC50 value indicated a more potent inhibition of cell growth and a better therapeutic outcome of the tumor.

For predicting the immune checkpoint blockade (ICB) responsiveness of two glioma groups, submap (Hoshida et al., 2007)^5^ was used to analyze the agreements of expression profile between the glioma set and reference set according to a published therapeutic data of ICB in melanoma (Roh et al., 2017).

### 2.10. Statistical analysis

All calculations were performed using the R software version 4.1.0. Kaplan–Meier (K-M) analysis and log-rank statistical tests were used to compare the groups’ overall survival (OS). Wilcoxon test was used to compare the differences within each clinicopathological subtype, immune cell infiltration, ssGSEA score, and chemotherapy reactivity. Fisher’s method was used to test the difference in response to ICB treatment. *p*-value <0.05 was considered statistically significant, whereby *p* < 0.05 was labelled as *, *p* < 0.01 as **, *p* < 0.001 as ***, and *p* < 0.0001 as ****.

## 3. Results

### 3.1. Invasive histology appears in mouse glioma with ITN

**Figure 1:**
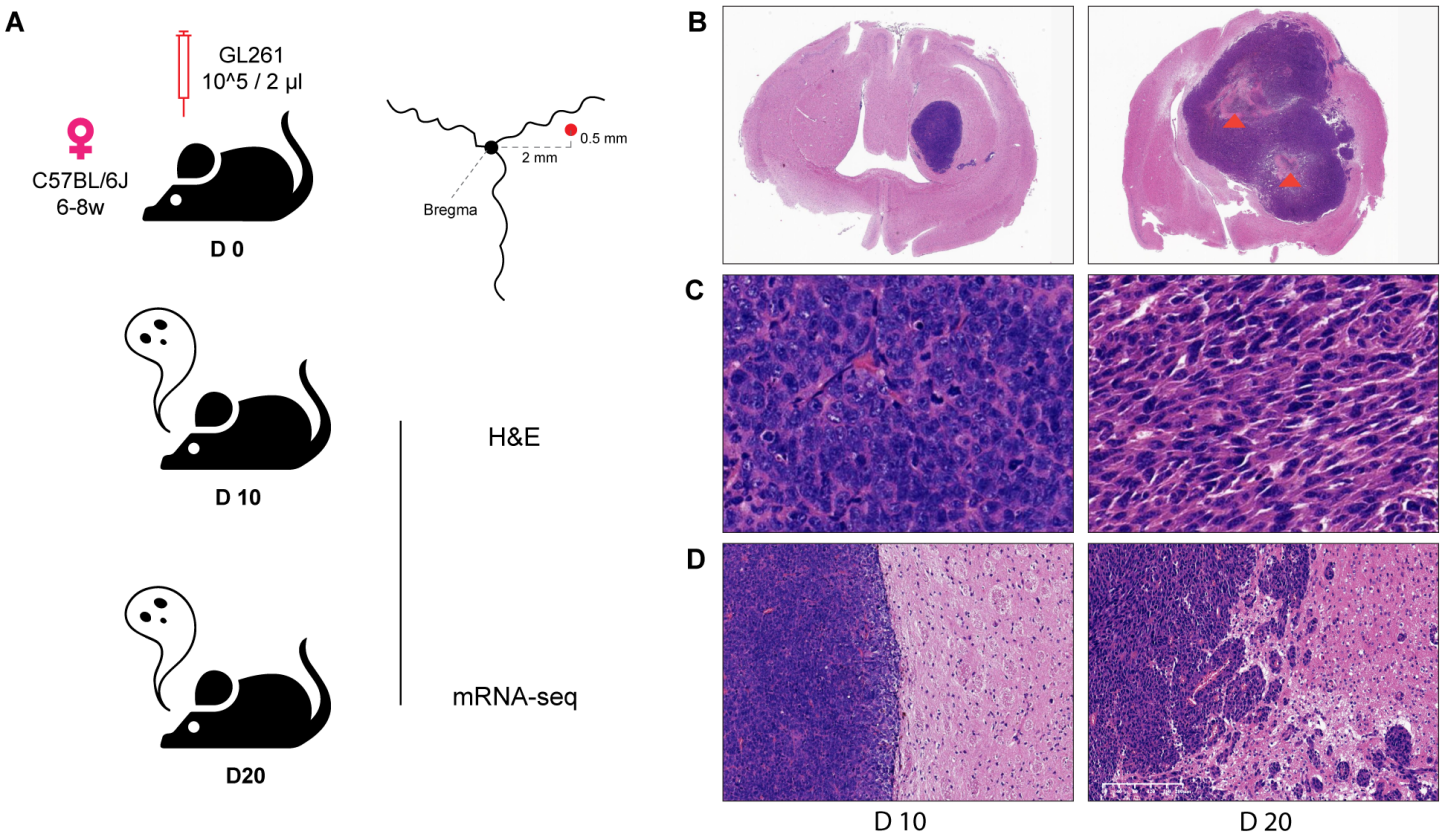
Histological difference between mouse glioma with and without ITN. **(A)** Diagram and parameters of mouse model composition and manipulation. **(B)** A broad view of glioma on day 10 (left, without ITN) and day 20 (right, with ITN) (1X), necrosis was mentioned by arrowhead (orange). **(C)** The parenchyma of glioma on day 10 and day 20 (40X). **(D)** Tumor border of glioma on day 10 and day 20 (10X).

According to our constructed models, GL261-C57BL/6 J syngeneic showed no ITN within 14 days after inoculation, while ITN reliably appeared on day 20. For excluding the incidence of ITN in non-necrotic glioma (NNG), day 10 (D10) was used as the specimen day of NNG and day 20 (D20) as the day of necrotic glioma (NG) (Figure 1A).

Histological features were observed through H&E staining of the Formalin-Fixed Paraffin-Embedded (FFPE) brain section of NNG and NG. Investigation of the whole brain (Figure 1B) showed no ITN in D10 (left), while extensive ITN was observed in D20 (right). Assessment of the tumor parenchyma (Figure 1C) revealed evenly distributed tumor cells with a round-like nucleus in D10. Conversely, disordered spindle-shaped cells with elongated nuclei were seen in D20. At the tumor border (Figure 1D), a clear boundary line was found to split between the tumor and brain tissue in D10. However, in D20, the disturbed boundary was captured, and a variety of small tumor cell islands were distributed at different distances from the tumor’s main body.

Herein, we illustrate a contrast between glioma without ITN and the apparent invasive histology in glioma with ITN.

### 3.2. Differentially expressed genes were characteristically enriched in cytokine-associated gene sets

After observing drastic differences in histology between the two groups, the whole tumor was used to characterize the expressive responses of the tumor after ITN. From a total of 30,177 genes detected by RNA-seq, 1,802 genes were significantly and differentially expressed (|FC| > 2, *q* < 0.05), with 685 up-regulated and 1,117 down-regulated genes identified on D20 compared to D10 (Supplementary Figure S1). Then, for capturing the representative genes after ITN, GO enrichment analysis was performed on the up-regulated and down-regulated differentially expressed genes (DEGs) in terms of biological process (BP), molecular function (MF), and cellular component (CC).

**Figure 2:**
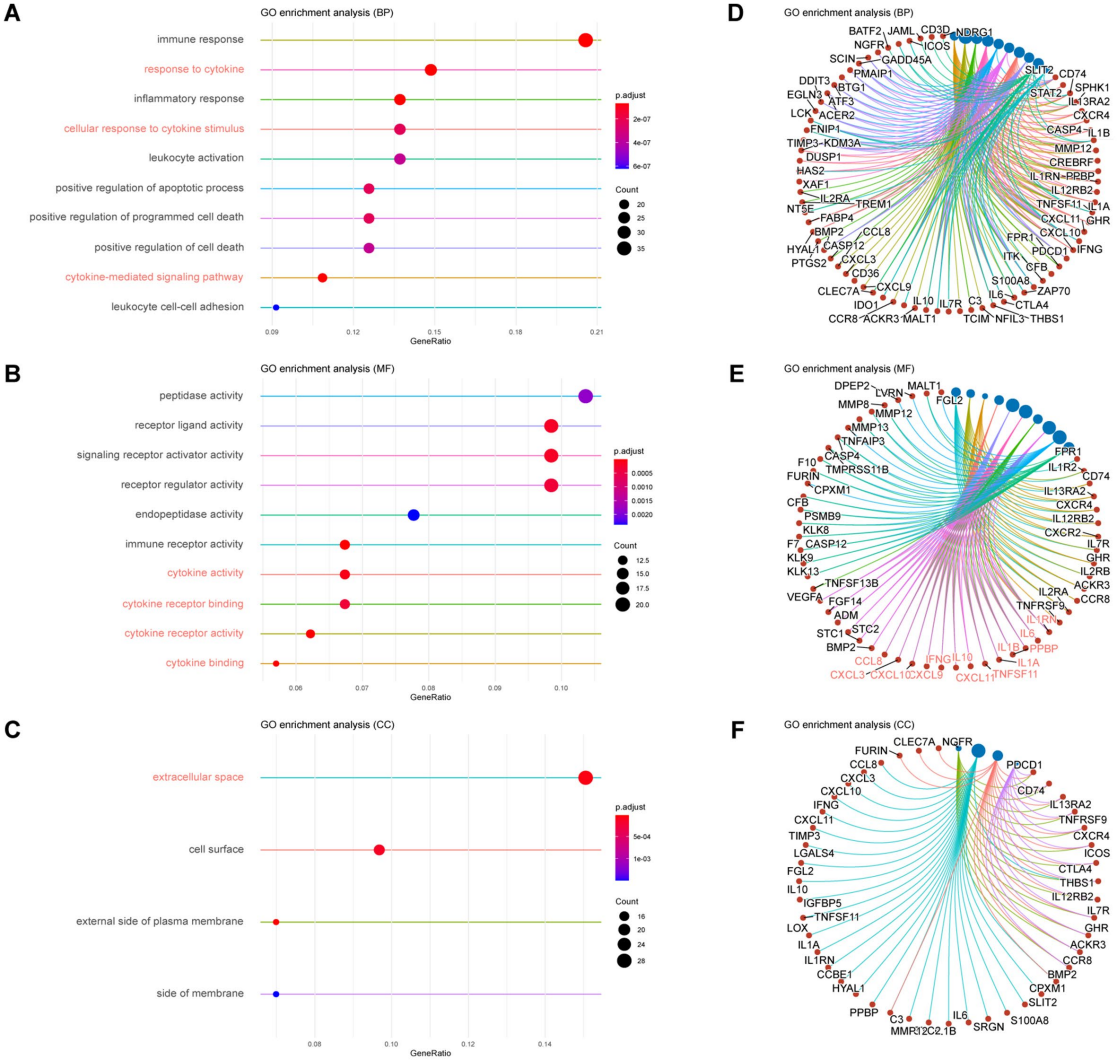
GO Enrichment of up-regulated DEGs in mouse glioma with ITN versus without ITN. **(A,D)** The top ten enriched biological processes with adjusted value of *p* (p.adjust) ranged by gene ratio **(A)** and individual genes of each process set **(D)**. **(B,E)** The top ten enriched molecular functions with p.adjust ranged by gene ratio **(B)** and individual genes of each function set **(E)**. **(C,F)** The top enriched cellular components with p.adjust >0.01 **(C)** and individual genes of each component set **(F)**.

Our results showed that seven of the top ten ranged BPs were related to immunity, and the other three were related to cell death in the enrichments of up-regulated DEGs. Of the seven enriched immune-related BPs, three were associated with a cytokine, namely, response to cytokines (GO: 0034097), cellular response to cytokine stimulus (GO: 0071345), and cytokine-mediated signaling pathway (GO: 0019221) (Figures 2A,D). In the top ten enriched MFs, except for two MFs related to peptidase activity, eight MFs were related to ligand and receptor activity, and four were associated with a cytokine, which were cytokine activity (GO: 0005125), cytokine receptor binding (GO: 0005126), cytokine receptor activity (GO: 0004896), and cytokine binding (GO: 0019955) (Figures 2B,E). While in the four CCs with significance, the extracellular space (GO: 0005615) was the most enriched, while the other three were related to the cell membrane (Figures 2C,F).

In the enrichments of down-regulated DEGs, all the top 10 enriched BPs were related to regular neuronal activity, such as the generation of neurons (GO: 0048699), synaptic signaling (GO: 0099536), and chemical synaptic transmission (GO: 0007268) (Supplementary Figures S2A,D). Ten enriched MFs functioned for synaptic signal transduction, such as transmembrane signaling receptor activity (GO:0004888), ion transmembrane transporter activity (GO:0015075), and inorganic molecular entity transmembrane transporter activity (GO:0015318) (Supplementary Figures S2B,E). Top enriched CCs were distributed in the cell membrane and synaptic locations, such as synapse (GO:0045202), an intrinsic component of the plasma membrane (GO:0031226), and an integral part of the plasma membrane (GO:0005887) (Supplementary Figures S2C,F).

The enrichment analysis presented an up-regulation of inflammatory activities that featured cytokine-related activities accompanied by cell death and peptidase. We also observed that down-regulated genes were enriched in normal neuronal activities.

### 3.3. ITN-associated cytokine pattern was highly expressed in mouse and human necrotic glioma

As cytokine is the key component of cytokine-related frameworks, genes enriched in cytokine activity (GO:0005125) were selected to determine the ITN-associated cytokine pattern. The 13 genes enriched in this gene set were Ccl8, Cxcl10, Cxcl11, Cxcl3, Cxcl9, Ifng Il10, Il1a, Il1b, Il1rn, Il6, Ppbp, and Tnfsf11.

**Figure 3:**
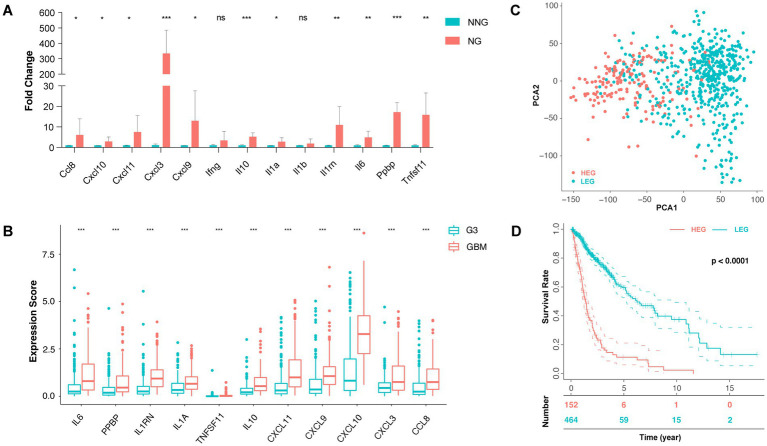
ITN-associated cytokine pattern and prognosis of related clusters. **(A)** qPCR of 13 genes enriched in cytokine activity (GO: 0005125) between necrotic glioma (NG) and non-necrotic glioma (NNG). **(B)** The expression score of 11 genes with significance was between GBM and grade 3 glioma. **(C)** Principal component analysis (PCA) of the high expression group (HEG) and the low expression group (LEG). **(D)** The 0-, 5-, 10-, and 15-year survival rates of HEG and LEG (* *p* < 0.05, ** *p* < 0.01, and *** *p* < 0.001).

Following the validation of these genes in mice, NG compared to NNG by qPCR, gene Il1b, and Ifng were excluded due to no statistical significance (*p* > 0.05). The genes (mean fold change) with *p* < 0.05 were Cxcl8 (6.11), Cxcl10 (2.85), Cxcl11 (7.50), Cxcl9 (13.00), and Il1a (2.81). Genes with *p* < 0.01 were Il1rn (10.97), Il6 (4.90), and Tnfsf11 (15.83), and those with *p* < 0.001 were Cxcl3 (334.5), Il10 (5.23) and Ppbp (17.20) (Figure 3A). In addition, we found that there were 11 cytokine genes highly expressed in glioma with ITN and committed in the ITN-associated cytokine pattern.

For clinicopathological diagnosis, necrosis is usually used to distinguish GBM (grade 4) from anaplastic glioma (grade 3). Thus, we set GBM as a necrotic group and compared it to grade III glioma as a non-necrotic group to further verify the ITN-associated cytokine pattern’s of the 11 genes determined from the TCGA database. In addition, 11 cytokine genes were compared between the two groups and were found to be significantly up-regulated in the necrotic group (*p* < 0.001) (Figure 3B).

Thus, we obtained an eleven-gene ITN-associated cytokine pattern.

### 3.4. Higher expression of ITN-associated pattern was related to shorter survival

Depending on the expression of the ITN-associated cytokine pattern, we divided the TCGA glioma samples into different group sets using the Consensus Clustering method. The consensus Clustering Plus algorithm showed that the two-member group was the best classification choice (Supplementary Figures S3A−D), based on which all the samples were divided into the HEG or the LEG group. Principal component analysis (PCA) revealed a differential distribution between the two groups (Figure 3C).

Further, the survival status of patients was compared between HEG versus LEG, which showed that the two-year survival rate was 22.37% (34/152) versus 48.06% (223/464), the five-year survival rate was 3.95% (6/152) versus 12.72% (59/464), the median survival rate was 1.09 years versus 1.90 years, and the mean survival rate was 1.52 years versus 2.72 years, respectively. Thus, the overall survival rate of the HEG groups was significantly lower than LEG (*p* < 0.0001) (Figure 3D). The prognostic value of individual genes was assessed in the TCGA glioma dataset. The results showed that CXCL3 was not significant (*p* = 0.982), while IL1A, IL1RN, IL6, IL10, TNFSF11, PPBP, CCL8, CXCL9, CXCL10, and CXCL11 were identified to the prognostic factors in glioma patients (value of *p* of TNFSF11 = 0.038, and the value of *p* of other genes were < 0.001) (Supplementary Figures S4A−K).

### 3.5. Clinical statuses were differentially distributed between HEG and LEG

Based on the vast prognostic difference between HEG and LEG, we further analyzed the relationship of the pattern with the following clinical statuses: MGMT promoter status, 1p/19q codeletion, IDH status, gender, age, and grade.

**Figure 4:**
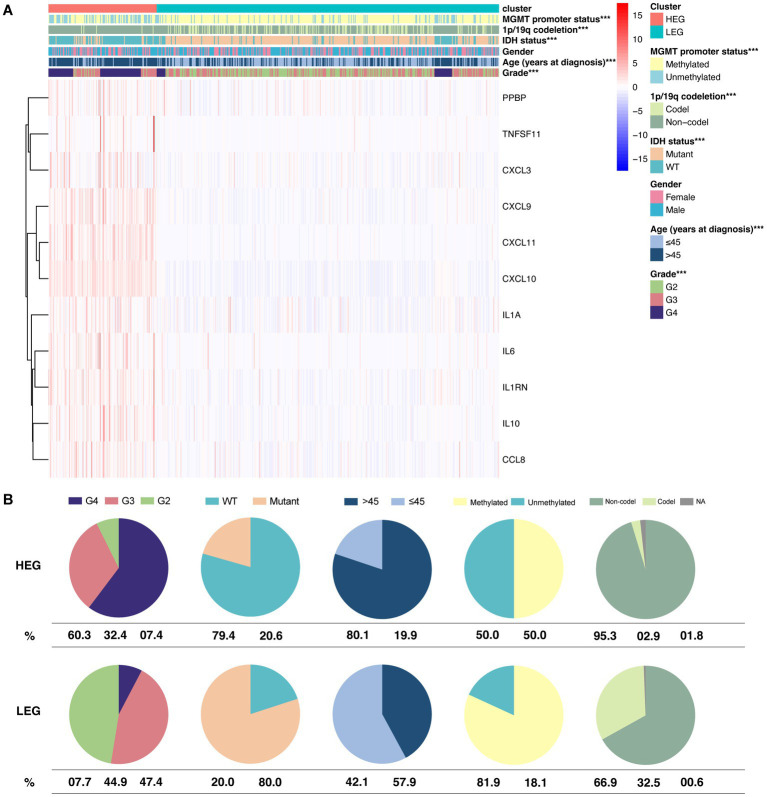
Differential distribution of clinical statuses between HEG and LEG. **(A)** The hot plot of 6 clinical statuses and expression of 11 HEG and LEG cytokine genes. **(B)** Pie plot showing the percentage of grade, IDH status, age group, MGMT promoter methylation status, and 1p/19q codeletion status in HEG and LEG (* *p* < 0.05, ** *p* < 0.01, and *** *p* < 0.001).

Our results showed that five of the six statuses were significantly different between the two groups (*p* < 0.01) (Figure 4A). Over half of the HEG were of grade 4 (60.3%). High-grade glioma (grade 3 and grade 4) comprised 92.6% of the HEG cohort. Comparatively, only 7.7% of the samples were grade 4, and nearly half (47.4%) were of grade 2 in the LEG cohort. In regard to IDH status, 79.4% of the HEG samples were wild-type (WT), while only 20% of the LEG samples were WT. Age > 45 years represented 80.1% of the HEG samples, while that of the LEG samples was only 42.1%. HEG samples had a lower detection rate (50%) of MGMT promoter methylation than the LEG samples (81.9%). Further, 1p/19q codeletion was seldom observed in the HEG samples (2.9%), while 32.5% of the LEG samples had 1p/19q codeletion (Figure 4B).

Although the expression of ITN-associated cytokine pattern was not related to gender, it was expressed in high-grade IDH-WT glioma and patients over 45 years old. In addition, we also observed that a high pattern expression was associated with a lower rate of MGMT promoter methylation and 1p/19q codeletion.

### 3.6. HEG possessed higher immune infiltration and immune checkpoint expression, but lower anti-tumour activity

Tumor-infiltrating immune cells and immune checkpoints play crucial roles in tumor biology, influencing tumor progression and response to therapy. In our study, we examined the immune characteristics of glioma with intra-tumoral necrosis (ITN) by evaluating immune infiltration, immune checkpoint expression, and anti-tumor activity in the HEG and LEG groups.

**Figure 5:**
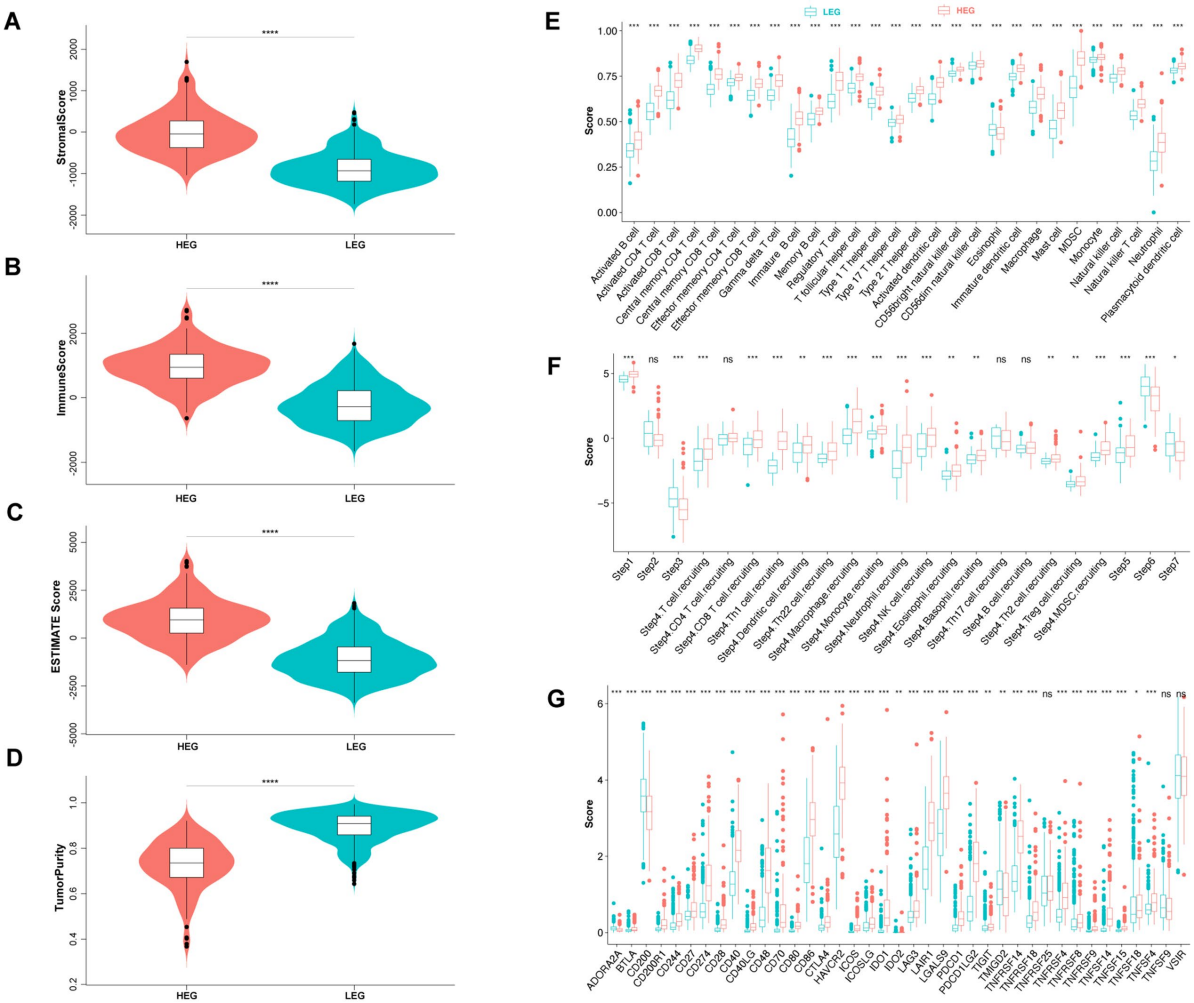
Comparison of tumor purity and immune status of HEG and LEG. **(A–D)** Stromal score, immune score, ESTIMATE score, and tumor purity of HEG and LEG. **(E)** Infiltration score of 28 immune cells between HEG and LEG. **(F)** Tumor immune cycle score of HEG and LEG. Step 1 (release of cancer antigen), step 2 (cancer antigen presentation), step 3 (priming and activation), step 4 (trafficking of immune cells to tumors), step 5 (infiltration of immune cells in tumors), step 6 (recognition of cancer cells by T cells) and step 7 (killing of cancer cells). **(G)** Immune checkpoint score of HEG and LEG (* *p* < 0.05, ** *p* < 0.01, *** *p* < 0.001, **** *p* < 0.0001).

Using the “ESTIMATE” algorithm, we assessed the stromal and immune scores of HEG and LEG samples. Consistent with the presence of ITN, HEG samples exhibited higher stromal and immune scores compared to LEG samples (*p* < 0.0001) (Figures 5A,B), indicating increased infiltration of immune cells and stromal components in the tumor microenvironment. This finding is in line with previous studies highlighting the role of inflammation and immune responses in tumor progression (Coussens and Werb, 2002; Bussard et al., 2016). ESTIMATE Score was determined with a combination of the two scores, and it was found to be higher in HEG than LEG (*p* < 0.0001) (Figure 5C). In addition, tumor purity was low in HEG compared to LEG (*p* < 0.0001) (Figure 5D).

Further analysis of immune cell infiltration revealed that HEG samples had higher infiltration scores for various immune cell types compared to LEG samples (Figure 5E). These immune cell populations included activated B cells, CD4 and CD8 T cells, dendritic cells, natural killer cells, and macrophages. The increased infiltration of these immune cell subsets suggests an activated immune response within the tumor microenvironment of HEG samples. This observation aligns with the pro-inflammatory cytokine profile observed in our study, suggesting a link between ITN, immune cell infiltration, and inflammatory processes in glioma.

Immune checkpoints, such as programmed cell death protein 1 (PD-1) and cytotoxic T-lymphocyte-associated protein 4 (CTLA-4), regulate the activity of immune cells and can impact tumor immune evasion (He and Xu, 2020). In our analysis, we found that HEG samples exhibited higher expression of immune checkpoints compared to LEG samples (Figure 5G). Specifically, several immune checkpoints, including CD271, CD40, CD86, HAVCR2, LAIR1, LGALS9, PDCD1LG2, were significantly upregulated in HEG samples. These findings suggest that glioma with ITN may employ immune checkpoint pathways to suppress anti-tumor immune responses and promote tumor growth.

Interestingly, despite the higher immune cell infiltration in HEG, our analysis revealed lower anti-tumor activity in this group (Figure 5F). The TIP tool, which evaluates different steps of the cancer immunity cycle, indicated that HEG samples had lower immune activation, tumor recognition, and killing of cancer cells compared to LEG samples. This discrepancy between immune cell infiltration and anti-tumor activity may be attributed to the upregulated expression of immune checkpoints in HEG, which could dampen the effector functions of infiltrating immune cells and hinder their ability to eliminate tumor cells.

In summary, our findings indicate that glioma with ITN (HEG) possesses higher immune infiltration, elevated expression of immune checkpoints, but lower anti-tumor activity. These immune characteristics suggest an immunosuppressive tumor microenvironment in HEG samples, potentially driven by the upregulated expression of immune checkpoints.

### 3.7. Chemotherapy and ICB were predicted to be more effective in HEG

**Figure 6:**
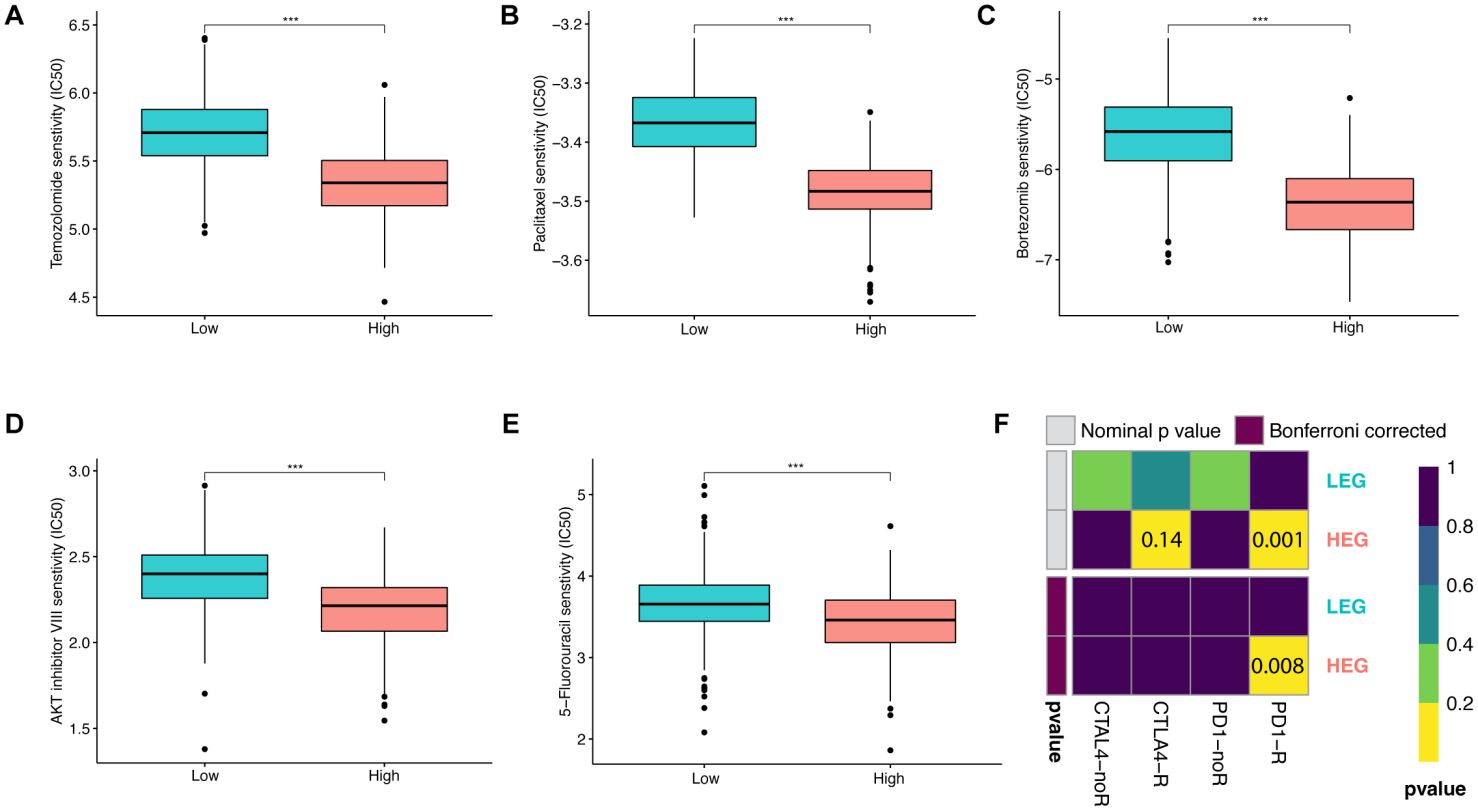
Predictive therapeutic responsiveness of HEG and LEG. **(A–E)** Predictive half-maximal inhibitory concentration (IC50) of Temozolomide, Paclitaxel, Bortezomib, AKT inhibitor VIII, and 5-Fluorouracil in HEG and LEG. **(F)** Predictive ICB responsiveness of CTLA4 and PD1 in HEG and LEG (* *p* < 0.05, ** *p* < 0.01, and *** *p* < 0.001).

After evaluation of the significant differences in the above aspects between HEG and LEG, the related predictive therapeutic effect was evaluated. Five chemotherapeutic drugs, namely, Temozolomide, Paclitaxel, Bortezomib, AKT inhibitor VIII, and 5-Fluorouracil, were tested with half-maximal inhibitory concentration (IC50). All the drugs were found to be more effective in HEG (*p* < 0.001) (Figures 6A–D). In the effectiveness of CTLA4 and PD1 blockade prediction, PD1 blockade was effective (Nominal *p* value = 0.001, Bonferroni corrected *p* value = 0.008) while CTLA4 was not effective in HEG (both value of *p* > 0.05) (Figure 6E). Thus, based on these results, the HEG group was predicted to be more responsive to chemotherapy and ICB than the LEG group.

## 4. Discussion

This study investigated the malignant histological transformation and inflammatory expression shift from the GL261 mouse glioma model without ITN to an ITN model. Genes related to cytokine activities were enriched to represent glioma with ITN from up-regulated DEGs. Eleven verified cytokine genes were highly expressed in both mouse and human glioma with ITN. Comparison between HEG and LEG showed that ITN-associated cytokine pattern in TCGA was associated with shorter survival, higher tumor grade, different clinical statuses, higher infiltration of inflammatory cells, higher immune checkpoint expression and favorable therapeutic effects in HEG.

The study of intra-tumoral necrosis (ITN) in glioma requires a reliable *in vivo* model presenting histological necrosis similar to that in clinical pathology. In this research area, such a model was lacking until 2022, a review published in *Acta Neuropathologica* by Markwell et al. described the following situation: “While TME restructuring following necrosis in glioblastoma appears to be an initiator of rapid tumor growth, appropriate animal models to establish the causal relationship between necrosis, TME alterations, and radial expansion are lacking (Markwell et al., 2022).” The widely used glioma model was established by the human glioma cell line implanted in nude mice. Previous research revealed that neither primary nor secondary glioblastoma cells, both with necrosis in pathology, developed ITN after implantation in mice (Lee et al., 2019). In the mouse cell line established glioma model, most models displayed the histology of astrocytoma (e.g., CT2A). Only the GL261-C57 model displayed glioblastoma histology, which is the presentation of ITN (Oh et al., 2014). Both in a previous research (Cha et al., 2003) and this present study (Figure 1), the GL261-C57 mouse model was confirmed to be a reliable model presenting ITN. This immunocompetent model provides a complete illustration of immune status in tumor biology. Thus, the GL261-C57 mouse model was the only choice for our research. This work focused on the ITN and ITN-related changes, which was achieved by comparing the glioma stage with ITN and the stage without ITN. Thus, the data reliability was not influenced by the number of cell lines included.

The invasive histological transformation was observed in the H&E staining of mouse glioma. On day 10 after GL261 cell inoculation, no sign of ITN was observed inside the tumor, which demonstrated evenly distributed round-nucleated cells and a clear boundary line enclosing the tumor from the normal brain. Comparatively, on day 20, profound ITN was observed in the center of the tumor, with disordered spindle-shaped cells directing in different directions, ruptured boundary lines and disseminated tumor islands. In the rat syngeneic model carrying C6 rat glioma cell, the tumor boundary was lost and pseudo-palisading necrosis appeared at the ischemic area after occlusion of the common carotid artery (Mamun et al., 2009). A mouse xenograft model carrying human glioma cells demonstrated invasion features at the tumor boundary without ITN after treatment of HMGB1, a DAMPs released from dead cells. In contrast, HMGB1 elevated tumor cell mobility *in vitro* (Li et al., 2019). Collectively, ITN appeared as the beginning of invasive transformation instead only as the result of malignant proliferation. The invasiveness of the tumor cells was associated with the molecules released by ITN.

Representative cytokine-related gene sets were enriched from DEGs when comparing mouse glioma with ITN to glioma without ITN. Most of the top enriched gene sets of BP, MF and CC were related to immune response and signal transduction in the extracellular space. Cytokine-associated sets were the most enriched. The primary function of cytokines is to regulate inflammation, especially immune response (Arango Duque and Descoteaux, 2014). Meanwhile, inflammation was long recognized as a prominent factor in tumor initiation, progression and metastasis (Coussens and Werb, 2002). Thus, the impact of ITN was not only on the activity of tumor cells but also on tumor immunity through the regulation of the expression of cytokines.

Eleven of the 13 up-regulated cytokine genes in RNA-sequencing were verified in both qPCRs of mouse glioma and human glioma databases. HEG samples carrying a higher expression of ITN-associated cytokine pattern demonstrated an unfavorable prognosis. In 11 cytokine genes, 9 of them were pro-inflammatory, including Il1a, Il6, Tnfsf11 and six chemokines, Ccl8, Cxcl3, Cxcl7(PPBP), Cxcl9, Cxcl10 and Cxcl11. It is worth noting that the threshold of the individual up-regulated genes differed between mouse data and human data, although a similar trend was observed between the two datasets. In this situation, the sample-obtaining procedure for further analysis should be considered. The human glioma sample for RNA-seq was obtained from surgically resected specimens based on which the model for pathological diagnosis was constructed. The necrosis status for the sample was unknown, even if the other sample was confirmed based on the necrosis features by a pathologist. On the other hand, the mouse sample was confirmed to contain necrosis on week 2, while no necrosis was observed on week 1. Thus, the mouse model could provide deeper insights into ITN and determine necrosis-associated features.

The eleven verified up-regulated genes had various functions. IL1RN and IL10 have anti-inflammatory functions, IL1 promotes inflammation, carcinogenesis and anti-tumor immunity (Mantovani et al., 2019), IL6 is pro-tumoral by activating carcinogenesis and tumor outgrowth (Jones and Jenkins, 2018), and IL10 promotes cytotoxicity but inhibits anti-tumor activities (Ouyang and O'Garra, 2019). Normally, chemokine mediates immune cell chemotaxis and lymphoid cell development. In cancer, they can regulate tumor cell proliferation, invasion and metastasis (Nagarsheth et al., 2017). The expression of CXCL3 was found to be about a hundred times higher in glioma with ITN than without ITN (Figure 3A). CXCL3 binds to CXCR2, which is expressed in endothelial cells, various immune cells and cancer cells. It is associated with cell chemotaxis, cancer invasion and metastasis and cancer stem cell maintenance (Zhang et al., 2016; Reyes et al., 2021). However, the expression of CXCL3 was not related to the prognosis of glioma patients in the TCGA dataset, possibly because of the relatively lower expression level. CXCL7 promotes tumor growth via the CXCR1/2 axis (Grepin et al., 2014). The CXCL9, 10, 11/CXCR3 axis have both anti-tumor and pro-tumor activity. Paracrine regulates the migration, differentiation, and activation of Th1 cells, further stimulating cytotoxic T lymphocytes and natural killer cells to perform anti-tumor action. Autocrine was shown to promote tumorigenesis, tumor proliferation and metastasis (Tokunaga et al., 2018). Taken together, the 11 overexpressed cytokines associated with ITN were pro-tumoral as they could activate tumor proliferation, invasion and metastasis and regulate immune infiltration, activation and inhibition.

Clinical statuses were differentially distributed between HEG and LEG, clustered with TCGA glioma samples. As HEG was composed predominantly of grade 4 (60.3%) and grade 3 (32.4%), it could be confirmed that grade 4 was characterized by histological necrosis. Partial grade 3 clustered in HEG might imply necrosis missed by selective biopsy in clinical practice. Opposite to LEG, IDH-WT composed 79.4% of HEG, similar to the WHO report, which reported that about 90% of GBM were IDH-WT and presented extensive necrosis. In contrast, necrosis in IDH-mutant GBM was found to be rather limited (Louis et al., 2016). The favorable outcome of IDH-mutant glioma may be associated with a lower incidence of ITN (Yan et al., 2009). A higher distribution (80.1%) in the sample of elderly patients with HEG could be related to a higher coagulation potential and higher probability of vascular events (Mari et al., 2008). The percentage of MGMT promoter methylation and 1p/19q was found to be higher in LEG. The favorable prognosis of MGMT promoter methylated, or 1p/19q co-deleted samples may be associated with a lower incidence of ITN (Boots-Sprenger et al., 2013).

The tumor purity of HEG was significantly lower than LEG due to the inflammatory cell infiltration induced by ITN. Although antigen releasing and immune cell infiltration were higher in HEG, immune activation, tumor recognition and killing were lower in HEG, possibly due to a higher expression of IL-10 and ICP in HEG. A worse prognosis of HEG was partially contributed by the breakdown of the Cancer Immunity Cycle (Chen and Mellman, 2013). Better predictive ICB responsiveness of PD1 in HEG may associate with higher CD8 T cell infiltration and CXCL9-11 expression (Zou et al., 2016). However, better predictive chemotherapy responsiveness in HEG contradicted previous knowledge that MGMT silencing benefited temozolomide chemotherapy (Riemenschneider et al., 2010).

In summary, our study showed that ITN was associated with glioma invasion and characteristic cytokine pattern expression, worse prognosis, higher immune infiltration and ICP expression, and better predictive therapeutic responsiveness in glioma. For a deeper appreciation of these cytokines and their role in glioma with ITN, the expression status of each cytokine in individual cell types should be confirmed in future studies.

## Data availability statement

The original contributions presented in the study are publicly available. This data can be found here: https://www.ncbi.nlm.nih.gov/sra/, PRJNA911267.

## Ethics statement

The animal study was reviewed and approved by the Animal Ethical and Welfare Committee of Zhejiang Provincial People’s Hospital.

## Author contributions

SH, HJ, HZ, and JD conceived and designed the study. HZ, JD, JZ, and NW performed experiments and collected the data. ZL, XY, and FW provided analytical and technical support, and participated in producing charts and pictures. HZ and JD drafted the manuscript. SH and HJ revised the manuscript. All authors contributed to the article and approved the submitted version.

## Funding

This work was funded by the National Natural Science Foundation of China (No. 61575058) and the Zhejiang Provincial People’s Hospital Talent Introduction Project (No. C-2021-QDJJ03-01).

## Conflict of interest

The authors declare that the research was conducted in the absence of any commercial or financial relationships that could be construed as a potential conflict of interest.

## Publisher’s note

All claims expressed in this article are solely those of the authors and do not necessarily represent those of their affiliated organizations, or those of the publisher, the editors and the reviewers. Any product that may be evaluated in this article, or claim that may be made by its manufacturer, is not guaranteed or endorsed by the publisher.

## Footnotes

^1^http://www.bioconductor.org/packages/release/bioc/html/DESeq2.html

^2^https://hiplot.com.cn/

^3^https://portal.gdc.cancer.gov/

^4^http://biocc.hrbmu.edu.cn/TIP/

^5^http://cloud.genepattern.org/gp

## Supplementary material

Supplementary Figure 1 Volcano plot of differentially expressed genes (DEGs). Gene with |Log2(FC)|>1 and *q*<0.05 was considered significant.

Supplementary Figure 2 GO Enrichment of down-regulated DEGs in mouse glioma with ITN versus glioma without ITN. **(A,D)** The top ten enriched biological processes with adjusted p-value (p.adjust) ranged by gene ratio **(A)** and individual genes of each process set **(D)**. **(B,E)** The top ten enriched molecular functions with p.adjust ranged by gene ratio **(B)** and individual genes of each function set **(E)**. **(C,F)** The top 10 enriched cellular components with p.adjust ranged by gene ratio **(C)** and individual genes of each component set **(F)**.

Supplementary Figure 3 Consensus clustering of TCGA glioma data based on ITN-associated cytokine pattern. **(A,B)** Consensus matrix of the 2-member group versus the 3-member group. **(C,D)** CDF plot and relative change in area under CDF curve.

Supplementary Table 1 Primer information of the 13 genes enriched in cytokine activity (GO: 0005125).
