# Supplementary figures and images for: Expression of intra-tumoral necrosis-associated cytokine pattern correlated with prognosis and immune status in glioma
Source: Front Mol Neurosci. 2023 Jul 3;16:1117237. doi: 10.3389/fnmol.2023.1117237 (PMC10352027; doi:10.3389/fnmol.2023.1117237)

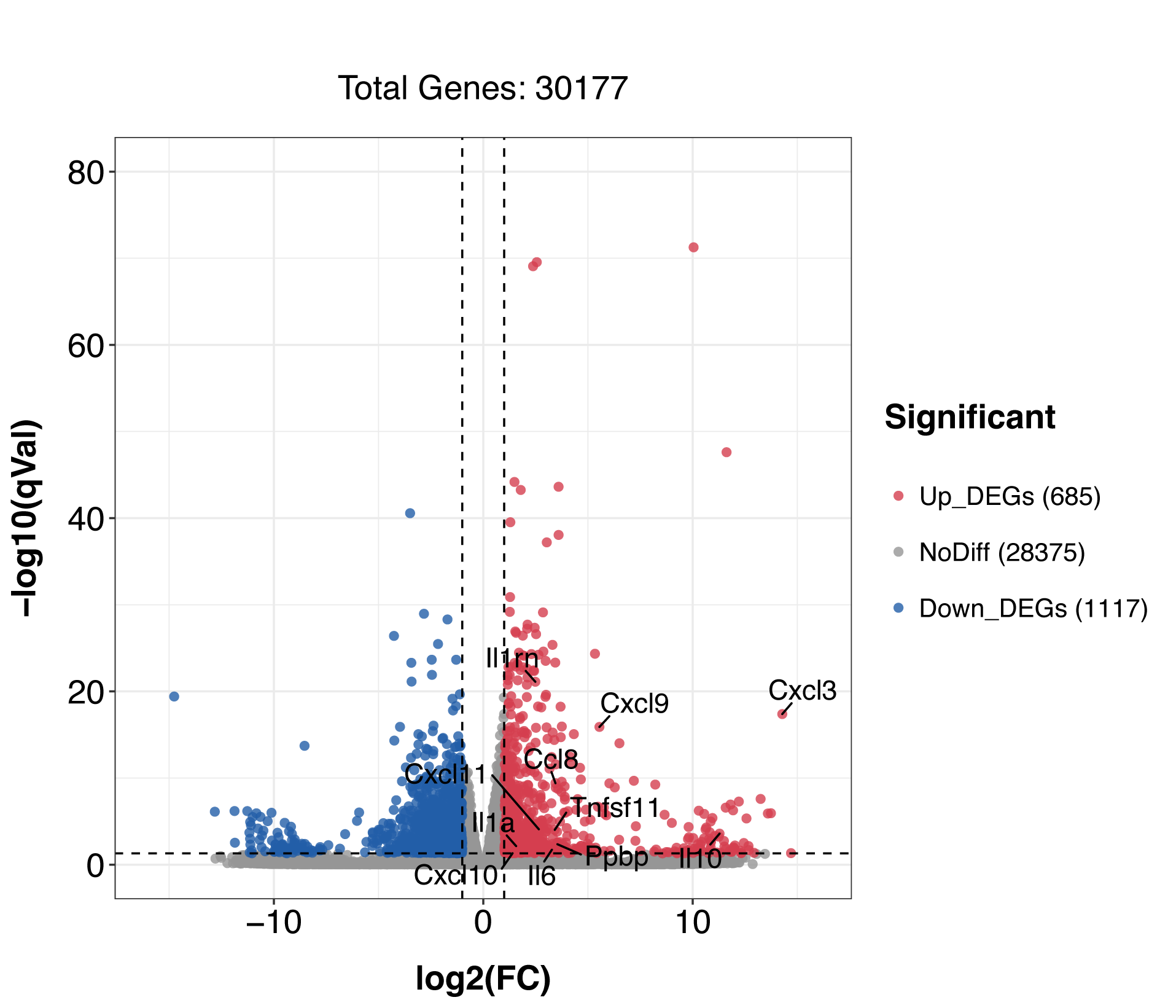

Supplement: Supplementary Figure 1 — Volcano plot of differentially expressed genes (DEGs). Gene with |Log2(FC)|>1 and q<0.05 was considered significant. [file Image_1.TIF]

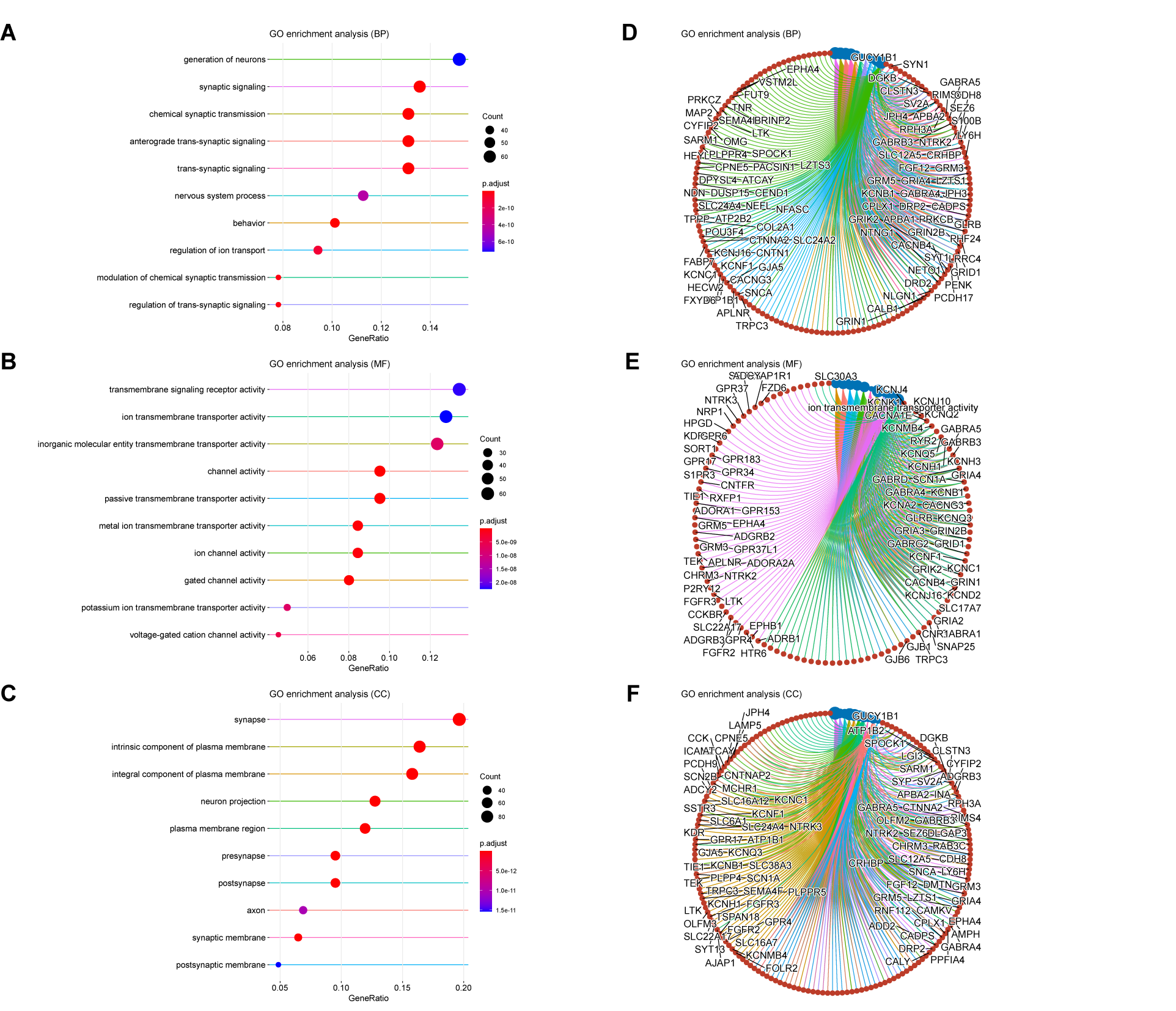

Supplement: Supplementary Figure 2 — GO Enrichment of down-regulated DEGs in mouse glioma with ITN versus glioma without ITN. (A,D) The top ten enriched biological processes with adjusted p-value (p.adjust) ranged by gene ratio (A) and individual genes of each process set (D). (B,E) The top ten enriched molecular functions with p.adjust ranged by gene ratio (B) and individual genes of each function set (E). (C,F) The top 10 enriched cellular components with p.adjust ranged by gene ratio (C) and individual genes of each component set (F). [file Image_2.TIF]

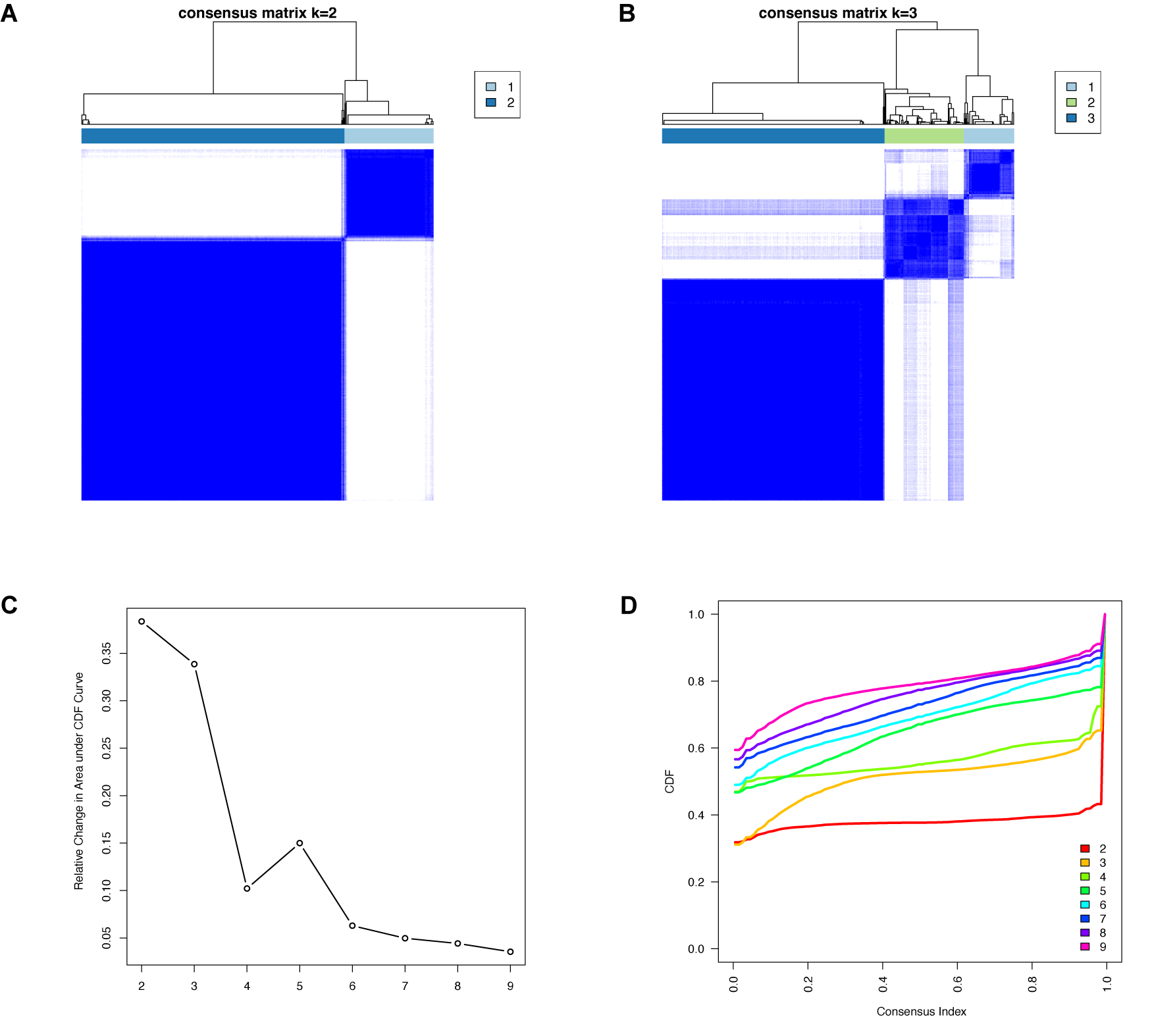

Supplement: Supplementary Figure 3 — Consensus clustering of TCGA glioma data based on ITN-associated cytokine pattern. (A,B) Consensus matrix of the 2-member group versus the 3-member group. (C,D) CDF plot and relative change in area under CDF curve. [file Image_3.TIF]

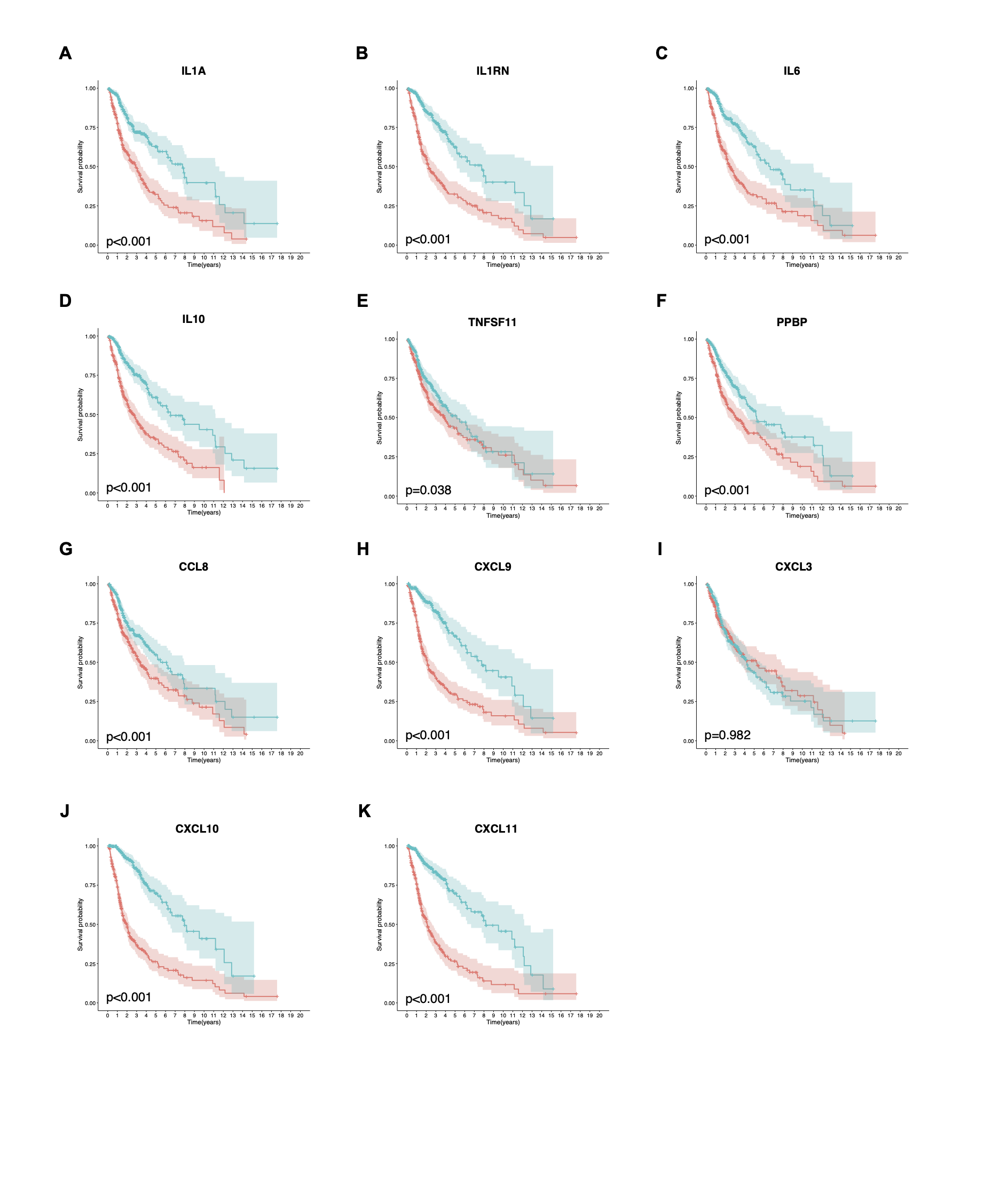

Supplement: Supplementary file 5 [file Image_4.TIFF]
